# Heat shock proteins (Hsp70 and Hsp90) in neurodegeneration: pathogenic roles and therapeutic potential

**DOI:** 10.3389/fnagi.2026.1711422

**Published:** 2026-02-12

**Authors:** Noureddine Ben Khalaf

**Affiliations:** Department of Molecular Medicine, College of Medicine and Health Sciences, Arabian Gulf University, Manama, Bahrain

**Keywords:** alpha-synuclein, heat shock proteins, molecular chaperone, neurodegenerative diseases, proteostasis, tau

## Abstract

The maintenance of protein homeostasis is essential for neuronal survival and function; however, it progressively declines with age, predisposing the brain to neurodegenerative diseases. Molecular chaperones Hsp70 and Hsp90 are key guardians of proteostasis, pivotally regulating protein folding, refolding, and degradation under both physiological and stress conditions. This review integrates an overview of the structural features, isoforms, and mechanistic interactions of Hsp70 and Hsp90. It highlights how their dysfunction contributes to the pathogenesis of major neurodegenerative disorders, including Alzheimer's disease, Parkinson's disease, amyotrophic lateral sclerosis, and Huntington's disease. We first examine the architecture and ATP-driven chaperone cycles of Hsp70 and Hsp90, their co-chaperone networks, and the feedback regulation by the Heat Shock Factor-1 pathway. We then discuss evidence linking age-related declines in chaperone expression and HSF-1 activity to proteostasis collapse and neuronal vulnerability. The review particularly examines how Hsp70 and Hsp90 differentially influence pathogenic protein aggregation (e.g., tau, α-synuclein, TDP-43, and mutant huntingtin) and how this balance is altered in the aging brain. Regarding therapeutic approaches, we summarize current strategies targeting these chaperones, including small-molecule modulators of Hsp70 and Hsp90, co-chaperone inhibitors, and recombinant chaperone therapy, which has shown to restore proteostasis and cognitive function in experimental models. These emerging interventions underscore the dual nature of Hsp70/Hsp90 systems, acting as both protectors and potential contributors to neurodegeneration, depending on their regulation and interaction context. By linking molecular chaperone biology to aging and translational therapeutics, this review establishes a framework for developing precision approaches that enhance proteostasis capacity, delay age-associated neurodegeneration, and promote healthy brain aging.

## Introduction

Molecular chaperones are critical proteins that uphold cellular proteostasis by facilitating the proper folding, assembly, and transport of newly synthesized or stress-denatured proteins (Gu et al., 2025). They inhibit the aggregation of misfolded proteins and facilitate their refolding or degradation, acting as a critical cellular defense mechanism against proteotoxicity (Singh et al., 2025a,b). The intricate network of chaperone-mediated pathways is crucial for cellular viability, particularly in post-mitotic cells such as neurons, where the buildup of misfolded proteins can lead to considerable functional impairments, notably neurodegeneration (Koszła and Sołek, 2024). Molecular chaperones play a critical role in protein quality control and are fundamental to comprehending the pathogenesis of neurodegenerative disorders linked to the accumulation of misfolded proteins (Koszła and Sołek, 2024). They serve as the primary defense mechanism of the cell against proteotoxicity; when proteins are unable to refold, chaperones direct them to degradation pathways, including the ubiquitin-proteasome system and autophagy, thus preventing harmful accumulation and reducing cellular damage (Müller and Hoppe, 2024).

This review outlines the roles of essential chaperone families representatives: Heat shock proteins Hsp70 and Hsp90, together with their complex interactions that regulate disease-related protein aggregation and neurotoxicity in major neurodegenerative diseases, such as Alzheimer's, Parkinson's, Amyotrophic Lateral Sclerosis, and Huntington's disease, emphasizing their function in reducing protein aggregation and cellular dysfunction. Additionally, this study will examine the therapeutic potential of targeting these chaperones, which may lead to innovative strategies for addressing these debilitating conditions.

While there is extensive literature on heat shock proteins in neurodegeneration, few reviews have integrated their roles within the broader context of age-related proteostasis decline. This review aims to bridge this gap by connecting the molecular biology of Hsp70 and Hsp90 to the gradual loss of chaperone capacity with age, and by exploring translational therapeutic strategies involving recombinant and pharmacological Hsp interventions. Consequently, it offers an updated synthesis that links molecular mechanisms with aging biology.

## The proteostasis network and neurodegeneration

In order to maintain cellular protein homeostasis, the proteostasis network, a complex system comprising chaperones, the ubiquitin-proteasome system, and autophagy, controls protein synthesis, folding, trafficking, and degradation (Lim and Vendruscolo, 2025). Molecular chaperones, key components of this network, recognize and bind to exposed hydrophobic regions of unfolded or misfolded proteins. This interaction prevents aggregation and aids in proper folding or directs proteins toward degradation pathways (Wankhede et al., 2022). This function is essential in neurons, which are particularly vulnerable to the accumulation of misfolded proteins due to their longevity and high metabolic demands (Lackie et al., 2017). Aberrant protein homeostasis in neurons, often due to impaired chaperone function, frequently leads to protein misfolding and aggregation, characteristic of various neurodegenerative disorders (Lackie et al., 2017; Wankhede et al., 2022). This vulnerability highlights the reason neurons are especially prone to genetic and environmental disturbances that impact proteostasis, resulting in a series of cellular malfunctions (Lackie et al., 2017). Consequently, comprehending the complex mechanisms through which chaperones preserve proteostasis and reduce protein aggregation is essential for elucidating the pathophysiology of neurodegenerative disorders and formulating effective therapeutic strategies (Lackie et al., 2017; Wankhede et al., 2022)

In fact, there is mounting evidence that the onset and progression of these debilitating conditions are significantly influenced by the dysregulation of molecular chaperones, making them promising therapeutic targets. A hallmark feature across many age-related neurodegenerative diseases, including Alzheimer's, Parkinson's, Huntington's diseases, and amyotrophic lateral sclerosis, is the aberrant accumulation of misfolded proteins, often presenting as amyloid deposits. This aggregation is frequently associated with aging, which is recognized as a primary risk factor for protein deposition diseases due to a progressive failure of the proteostasis network (Chiti and Dobson, 2017; Hipp et al., 2019). This age-related decline encompasses reduced chaperone levels and activity (Broadley and Hartl, 2009; Chiti and Dobson, 2017; Hinault et al., 2006), diminished ubiquitin-proteasome system function (Chiti and Dobson, 2017; Davidson and Pickering, 2023; Hinault et al., 2006), decreased efficiency of autophagy (Chiti and Dobson, 2017), and increased oxidative stress (Chiti and Dobson, 2017). These impairments collectively lead to the accumulation of toxic protein aggregates that compromise neuronal function and viability (Bobori et al., 2017; Ciechanover and Livneh, 2025; Johnson et al., 2025; Sharma et al., 2023). Several forms of aggregates were reported, including soluble oligomers and insoluble amyloid fibrils, both of which are implicated in cellular toxicity and disease progression (Johnson et al., 2025). In essence, the failure of the proteostasis network, particularly the chaperone system, to adequately manage protein folding and clear misfolded species is a core tenet in the pathogenesis of these neurodegenerative conditions. As a consequence, supporting molecular chaperone activity and/or restoring proteostasis could represent a potent strategy to counteract the pathogenic effects of protein aggregation in neurodegeneration.

In the following sections, we will delve into the role of essential Heat Shock Proteins; Hsp70 and Hsp90, in proteostasis and their critical functions and neuroprotective properties within the central nervous system, given their longstanding recognition as vital components of the cellular stress response.

## The role of Hsp70 and Hsp90 in proteostasis

**Figure 1:**
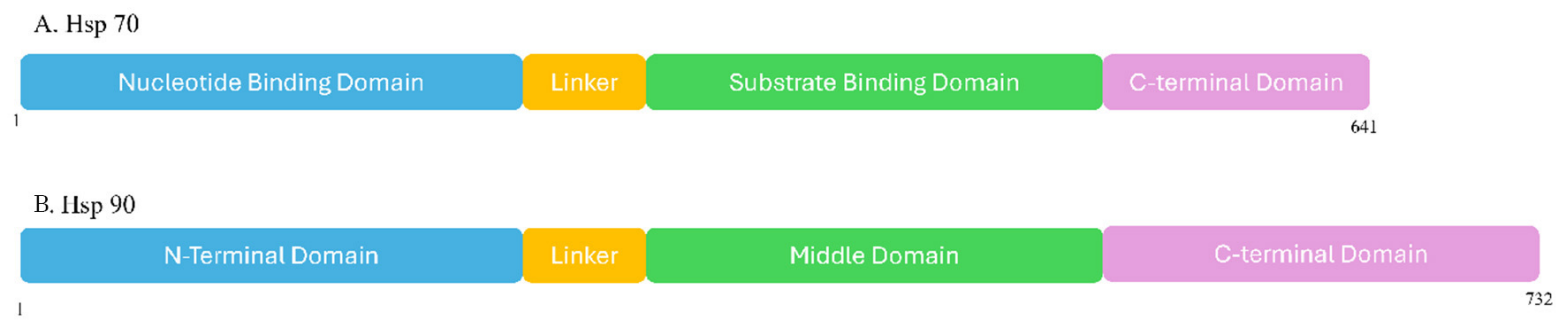
Domain organization of Hsp70 and Hsp90. **(A)** Hsp70 consists of an N-terminal nucleotide-binding domain (NBD) responsible for ATP binding and hydrolysis, which drives substrate association and release; a flexible interdomain linker that coordinates allosteric communication between the NBD and the substrate-binding region; and a C-terminal substrate-binding domain (SBD) comprising a β-sandwich subdomain and an α-helical lid that encloses unfolded client peptides. The extreme C-terminal tail often contains an EEVD motif critical for co-chaperone recognition. **(B)** Hsp90 contains an N-terminal ATP-binding domain (NTD) that initiates the chaperone cycle, a charged flexible linker region, a middle domain (MD) involved in client protein binding and ATP hydrolysis, and a C-terminal dimerization domain (CTD) harboring the conserved MEEVD motif for interaction with tetratricopeptide-repeat (TPR) co-chaperones such as Hop, FKBP51, and FKBP52.

Heat shock proteins are a superfamily of molecular chaperones classified by their molecular weight, with Hsp70 and Hsp90 as key members. These proteins are highly abundant in eukaryotic cells, particularly in neuronal cells, and essential for maintaining proteostasis under several conditions (Finka and Goloubinoff, 2013). Hsp70 is composed of two domains: a nucleotide-binding domain (NBD) and a substrate-binding domain (SBD) (Mayer and Bukau, 2005) (Figure 1A). This structural organization enables Hsp70 to bind to nascent polypeptides and partially folded client proteins, either preventing aggregation and facilitating proper protein folding or directing them toward degradation pathways (Choudhary et al., 2025). This ATP-dependent process is further regulated by co-chaperones, such as J-domain proteins (notably Hsp40), which modulate Hsp70's ATPase cycle and client protein specificity, thereby supporting its efficacy in protein quality control (Singh et al., 2025a,b). Operating often in concert with these co-chaperones, Hsp70 plays a critical role in early protein folding events and disaggregation (O'Connor et al., 2025).

Similarly, in eukaryotic cells, Hsp90 constitutes about 1% of total proteins. There are currently four known Hsp90 paralogs: Hsp90α and Hsp90β, which are found in the cytosol, TRAP1 in the mitochondria, and GRP94 in the endoplasmic reticulum. The maturation and stabilization of a variety of client proteins, such as transcription factors and signaling molecules, depend on Hsp90 (Wei et al., 2024). Functional Hsp90 proteins were reported to form (Somogyvári et al., 2022) active homodimers through the C-terminal domain (Wei et al., 2024) (Figure 1B). When ATP binds to Hsp90, it induces a conformational change to form the active dimer (Singh et al., 2025a,b). ATP hydrolysis then facilitates the chaperone function. In addition, several co-chaperones such as stress-inducible protein (STIP-1), cell division cycle 37 (CDC37), protein phosphatase 5 (PP5), FK506-binding protein 51 (FKBP51), FK506-binding protein 52 (FKBP52), and cyclophilin 40 (Cyp40), facilitate those transformations and client protein refolding process (Koszła and Sołek, 2024; Marzano et al., 2025).

According to Gupta et al. (2019), these ATP-dependent chaperones actively identify and interact with misfolded proteins in order to facilitate their refolding or temporarily sequester them to prevent aggregation. Their coordinated action is essential for cellular adaptation to a variety of stresses, preventing protein denaturation, and preserving overall cellular homeostasis (Saxena et al., 2025; Singh et al., 2025a,b). These chaperones' dysregulation or lack of activity can impair the cell's capacity to control misfolded proteins, which can result in the development and advancement of neurodegenerative diseases (Gupta et al., 2019). In fact, across a variety of neurodegenerative models, overexpression of Hsp70 and Hsp90 has been linked to a marked decrease in inflammation, protein aggregation, and neuronal loss (Gupta et al., 2019; Lackie et al., 2017).

The specific roles that Hsp70 and Hsp90, as well as their co-chaperones, play in reducing the pathological features of neurodegenerative diseases, like the aggregation of tau protein and β-amyloid in Alzheimer's disease, will be covered in more detail in the following sections (Batko et al., 2024). To explore their therapeutic potential, this discussion will also cover their roles in preventing alpha-synuclein aggregation in Parkinson's disease, addressing misfolded TDP-43 and SOD1 in Amyotrophic Lateral Sclerosis, and addressing mutant huntingtin in Huntington's disease (Batko et al., 2024; Lackie et al., 2017). Future sections will also critically assess the challenges inherent in translating chaperone-based therapies from preclinical models to clinical application.

## Structural features, isoforms, and localization of Hsp70 and Hsp90

Molecular chaperones exist in various isoforms, each exhibiting distinct subcellular localizations and specialized functions essential for maintaining proteostasis within different cellular compartments. The structural diversity, isoform specialization, and subcellular localization of Hsp70 and Hsp90 family members are summarized in Table 1.

**Table 1:** Structural features, isoforms, and cellular localization of Hsp70 and Hsp90.

**Protein**	**Major isoform (common name)**	**Gene name**	**Cellular localization**	**Stress inducibility**	**Key functional role**
Hsp70	Inducible Hsp70	HSPA1A/HSPA1B	Cytosol, nucleus	Inducible	ATP-dependent folding of nascent and misfolded proteins; suppression of toxic aggregation (tau, α-synuclein, huntingtin)
	Heat shock cognate 70 (Hsc70)	HSPA8	Cytosol, nucleus	Constitutive	Basal proteostasis, clathrin-mediated endocytosis, chaperone-mediated autophagy
	GRP78/BiP	HSPA5	Endoplasmic reticulum	Stress-inducible (UPR)	ER protein folding, unfolded protein response, ER stress regulation
	Mortalin/mtHsp70	HSPA9	Mitochondria	Constitutive	Mitochondrial protein import, oxidative stress resistance, neuronal survival
	Hsp70B′	HSPA6	Cytosol	Highly inducible	Acute stress response, neuronal protection under severe stress
Hsp90	Hsp90α	HSP90AA1	Cytosol, nucleus	Inducible	Folding and stabilization of signaling proteins and neurodegeneration-associated clients
	Hsp90β	HSP90AB1	Cytosol	Constitutive	Maintenance of basal proteostasis and chaperone buffering capacity
	GRP94	HSP90B1	Endoplasmic reticulum	Stress-responsive	ER proteostasis, folding of secretory and membrane proteins
	TRAP1	TRAP1	Mitochondria	Constitutive	Regulation of mitochondrial quality control, oxidative stress, and apoptosis

The Hsp70 family in humans comprises 13 distinct genes (HSPA1A, HSPA1B, HSPA1L, HSPA2, HSPA5, HSPA6, HSPA7, HSPA8, HSPA9, HSPA12A, HSPA12B, HSPA13, and HSPA14) that encode molecular chaperones with diverse expression patterns, cellular localizations, and stress inducibility. Within this family, functional diversity arises from compartment-specific isoforms that support protein quality control across distinct cellular environments. Constitutively expressed cytosolic Hsc70 (HSPA8) fulfills essential housekeeping roles, whereas inducible Hsp70 (HSPA1A) is rapidly upregulated during cellular stress to enhance proteostasis capacity (Ambrose and Chapman, 2021; Takakuwa et al., 2019). In the endoplasmic reticulum, GRP78 (HSPA5) governs protein folding and quality control through regulation of the unfolded protein response and ER-associated degradation pathways, while the mitochondrial isoform Mortalin (HSPA9) facilitates protein import and folding within the mitochondrial matrix in coordination with mtHsp60 (Ambrose and Chapman, 2021; Gu et al., 2025; Singh et al., 2025a,b). This compartmentalization and functional specialization, summarized in Table 1, enable precise, context-dependent regulation of proteostasis under both basal and stress conditions. In parallel, additional chaperone systems, including Hsp40, Hsp60, and small heat shock proteins, complement Hsp70/Hsp90 activity through distinct but cooperative mechanisms to maintain protein homeostasis (Batko et al., 2024; Sulistio and Heese, 2015).

Hsp70 chaperones are among the most conserved and ubiquitous components of the cellular proteostasis network, present from bacteria to humans and adapted to function across distinct subcellular compartments (Gu et al., 2025). This evolutionary conservation underlies a family of paralogs with specialized localizations—cytosolic, endoplasmic reticulum, and mitochondrial—allowing coordinated control of protein folding, trafficking, and degradation throughout the cell (Hervás and Oroz, 2020; Rutledge et al., 2022). In the cytosol, constitutively expressed Hsc70 (HSPA8) maintains basal proteostasis, supports clathrin-mediated endocytosis and chaperone-mediated autophagy, and interacts with a broad client repertoire, whereas inducible Hsp70 isoforms (HSPA1A/B) are transcriptionally upregulated by HSF-1 during stress to buffer proteotoxic insults (Rosenzweig et al., 2019; Venediktov Artem et al., 2023). Compartment-specific isoforms such as GRP78 in the endoplasmic reticulum and Mortalin in mitochondria further extend Hsp70 function to organelle-specific quality control (Table 1). Together, this functional diversification enables Hsp70 family members to counteract protein misfolding and aggregation across cellular contexts, a property central to their protective role in neurodegenerative disease (Chow et al., 2010; Gupta et al., 2019).

The structural organization of Hsp70 is defined by two primary domains: an N-terminal nucleotide-binding domain and a C-terminal substrate-binding domain, interconnected by a flexible linker (Figure 1A) (Ambrose and Chapman, 2021; Châari, 2019; Gupta et al., 2019; Koszła and Sołek, 2024; Takakuwa et al., 2019). The NBD is responsible for ATP binding and hydrolysis, which powers the chaperone's activity (Ambrose and Chapman, 2021; Gu et al., 2025; Gupta et al., 2019; Takakuwa et al., 2019). This domain is composed of subdomains that coordinate ATP binding and control the interaction with client proteins (Ambrose and Chapman, 2021; Gupta et al., 2019). The SBD recognizes and binds to unfolded or misfolded client proteins, particularly hydrophobic regions (Ambrose and Chapman, 2021; Gu et al., 2025; Gupta et al., 2019; Takakuwa et al., 2019). It comprises a β-sheet-rich base and an α-helical lid that functions to clamp down on the substrate (Ambrose and Chapman, 2021; Châari, 2019; Gu et al., 2025; Hartl et al., 2011; Koszła and Sołek, 2024). The flexible linker facilitates allosteric communication between the NBD and SBD, transmitting conformational changes during the ATP-dependent cycle (Hervás and Oroz, 2020; Koszła and Sołek, 2024; Takakuwa et al., 2019). Hsp70 alternates between an ATP-bound “open” state with low substrate affinity and an ADP-bound “closed” state with high substrate affinity, a process regulated by co-chaperones like Hsp40 and nucleotide exchange factors (Gu et al., 2025; Gupta et al., 2019; Hartl et al., 2011; Koszła and Sołek, 2024). The NBD's V-shaped structure, featuring subdomains Ia and IIa, engages with ATP, driving conformational changes essential for the chaperone mechanism (Koszła and Sołek, 2024). These dynamic alterations in the NBD, in conjunction with the SBD's ability to bind hydrophobic sequences, enable Hsp70 to regulate the folding and refolding of substrate proteins in an ATP-dependent manner (Châari, 2019; Gupta et al., 2019). This mechanism ensures the efficient processing of nascent polypeptides, facilitating their proper folding and preventing aggregation (Gu et al., 2025; Mitra and Chatterjee, 2024). However, HSP0's intrinsic ATPase activity is relatively low without a client, necessitating co-chaperones like J-domain proteins to stimulate ATP hydrolysis and channel client proteins to Hsp70 (Gupta et al., 2019; Maiti et al., 2014). The intricate interplay between Hsp70 and its co-chaperones, such as Hsp40 and nucleotide exchange factors, orchestrates a finely tuned ATPase cycle crucial for substrate binding and release (Hervás and Oroz, 2020; Wang et al., 2021). Specifically, the J-domain proteins (Hsp40s) stimulate the Hsp70 ATPase activity, facilitating client protein capture and preventing aggregation, while nucleotide exchange factors accelerate ADP release, allowing for timely ATP rebinding and subsequent substrate dissociation (Wang et al., 2021; Yang et al., 2017). This complex regulatory mechanism ensures that Hsp70 can efficiently bind to and release client proteins, modulating their folding status and preventing their aggregation in a highly coordinated fashion (Marszałek and Craig, 2022; Wang et al., 2021). This cyclical interaction of substrate binding, ATP hydrolysis, and subsequent release is crucial for promoting proper protein folding and preventing the accumulation of aberrant protein structures, which are implicated in various pathological conditions (Broadley and Hartl, 2009; Balchin et al., 2016).

The mammalian Hsp90 family comprises highly conserved molecules involved in myriad cellular processes. These isoforms, including Hsp90α, Hsp90β, GRP94, and TRAP1, exhibit distinct subcellular localizations and engage with specific client proteins, reflecting their specialized functions within the proteostasis network (Gupta et al., 2019). The distinct localization and substrate specificity of these Hsp90 isoforms enable them to play critical roles in mitigating protein misfolding associated with various neurodegenerative conditions (Bohush et al., 2019). Hsp0's structural organization is characterized by three main conserved domains: an N-terminal domain (NTD), a middle domain (MD) and a C-terminal domain (CTD) (Bohush et al., 2019) (Figure 1B). In eukaryotes, a variable charged linker domain connects the NTD and MD (Hoter et al., 2018). The NTD is responsible for ATP binding, while the MD interacts with client proteins and co-chaperones, containing a critical hinge region for substrate affinity (Singh et al., 2025a,b). The CTD facilitates the dimerization of Hsp90, forming a flexible homodimer essential for its function (Singh et al., 2025a,b).

Hsp90's chaperone activity is ATP-dependent, where ATP binding induces significant conformational changes, including the formation of a “molecular clamp” as the N-terminal domains dimerize. ATP hydrolysis then returns the chaperone to an open conformation (Hoter et al., 2018). This intricate ATP-driven conformational cycle is critical for Hsp90 to effectively engage with and mature a diverse array of client proteins, many of which are key regulatory components in cellular signaling pathways (Hervás and Oroz, 2020). The NTD is particularly noteworthy due to its highly conserved ATP-binding site, exhibiting 95% identity between HSP90α and HSP90β, with only two amino acid residue differences (Gu et al., 2025). This high degree of conservation underscores the functional importance of this domain in ATP-dependent chaperone activity across different cytosolic Hsp90 isoforms (Peng et al., 2022). The active unit of all Hsp90 paralogs is formed by the homodimerization of three distinct regions interconnected by flexible linkers (Gupta et al., 2019). Specifically, the NTD is accountable for nucleotide binding, the MD recognizes client proteins and triggers ATP hydrolysis, and the CTD is crucial for dimerization (Gupta et al., 2019). This dimeric assembly is further stabilized by various co-chaperones and ATP, which drive a sophisticated conformational cycle essential for client protein folding (Girstmair et al., 2019). The ATP-binding site within the NTD is indispensable for the chaperone cycle of Hsp90, with the MD being crucial for client binding and ATP hydrolysis, a process initiated by the interaction between the N-terminal ATP-binding site and the MD (Gu et al., 2025). This intricate interplay facilitates the “molecular clamp” mechanism, where ATP hydrolysis drives conformational dynamics essential for client protein remodeling and interactions (Blair et al., 2018). This dynamic process, tightly regulated by the ATP hydrolysis cycle, ensures that client proteins undergo the necessary conformational changes to achieve their mature, functional states (Ferraro et al., 2018). The NTD houses the ATP-binding pocket, sharing high conservation with the GHKL superfamily, and its ATP-binding site is critical for the chaperone cycle, being a primary target for inhibitors like geldanamycin and radicicol (Hoter et al., 2018). The MD of Hsp90 plays a pivotal role in modulating its ATPase activity by interacting with the γ-phosphate of ATP bound to the NTD; it also serves as a crucial binding site for co-chaperones like Aha1 and client proteins (Hoter et al., 2018). The CTD contains the MEEVD motif, which interacts with TPR-domain co-chaperones, and is crucial for the homodimerization of Hsp90 (Wang et al., 2021). In the absence of ATP, the Hsp90 homodimer adopts an open V-shaped conformation; however, ATP binding to the NTD induces significant structural rearrangements, leading to the closure of the N-terminal lids and subsequent dimerization of the NTDs (Wang et al., 2021). These structural transitions, driven by ATP binding, progressively lead to a closed state where the N-terminal domains associate with the middle domains, forming a compact structure conducive to client protein maturation and folding (Meyer et al., 2003).

Beyond their canonical roles, Hsp70 and Hsp90's intricate interactions with their co-chaperones facilitate a dynamic response to cellular stress, enabling a nuanced regulation of protein folding and degradation pathways (Batko et al., 2024; Singh et al., 2025a,b). For example, the co-chaperones can modulate the ATPase activity of Hsp70 and Hsp90, thereby influencing the rate and efficiency of client protein binding and release (Wang et al., 2021). This complex interplay forms a robust chaperone machinery crucial for cellular homeostasis and protein quality control (Gupta et al., 2019). Dysfunction within this proteostasis network, particularly involving Hsp70 and Hsp90, is a molecular hallmark of several neurodegenerative diseases, including Alzheimer's disease, Parkinson's disease, Huntington's disease, and amyotrophic lateral sclerosis, where protein aggregation is a common pathological feature (Lackie et al., 2017; Wang et al., 2021). Disturbances in this chaperone machinery lead to the accumulation of misfolded proteins, a key mechanism underlying age-related neurodegenerative disorders (Panda et al., 2023). Consequently, targeting the interactions between chaperones and co-chaperones presents a novel therapeutic strategy for mitigating the progression of these debilitating conditions by restoring proteostasis (Wang et al., 2021).

Building upon a comprehensive understanding of the intricate structural features, distinct isoforms, and specific subcellular localizations of Hsp70 and Hsp90, this review will now transition to their profound and evolving roles in the context of aging. The progressive decline in proteostasis with advancing age is a central driver of neurodegenerative diseases, and these molecular chaperones are instrumental in counteracting this vulnerability. The subsequent discussion will illuminate how the function and regulation of Hsp70 and Hsp90 are altered during the aging process, directly influencing cellular resilience and susceptibility to misfolded protein stress.

## Role of Hsp70 and Hsp90 in aging

**Figure 2:**
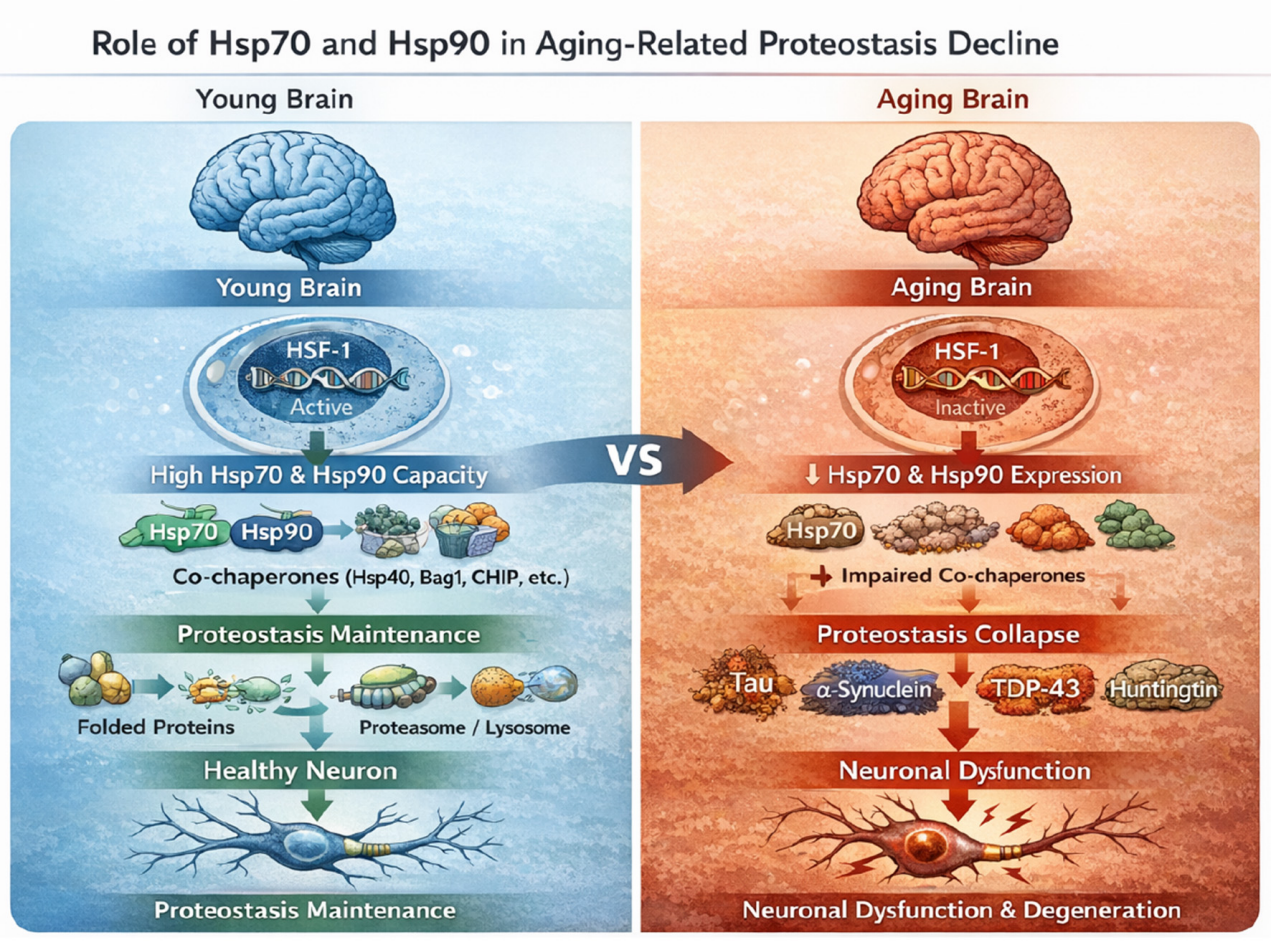
Role of Hsp70 and Hsp90 in brain aging. Schematic comparison of the young vs. aging brain. In the young brain, active HSF-1 supports robust Hsp70/Hsp90 expression and co-chaperone activity, enabling efficient protein folding and clearance through the proteasome/lysosome and maintaining neuronal homeostasis. In the aging brain, reduced HSF-1 activity lowers Hsp70/Hsp90 capacity and impairs co-chaperone support, leading to proteostasis collapse and accumulation of aggregation-prone proteins (tau, α-synuclein, TDP-43, huntingtin), ultimately contributing to neuronal dysfunction and degeneration.

During aging, the expression and activity of major heat shock proteins, including Hsp70 and Hsp90, progressively decline, leading to a reduced capacity for proteostasis and an increased vulnerability to misfolded protein stress (Broadley and Hartl, 2009; Hinault et al., 2006; Singh et al., 2025a,b; Venediktov Artem et al., 2023). This age-related decrease in chaperone function is evidenced by a decline in Hsp70 transcription in the human brain and a continuous reduction in the DNA binding activity of Heat Shock Factor 1 (HSF-1) in aged rat hepatocytes (Broadley and Hartl, 2009; Wentink et al., 2025). Consequently, the cell's ability to activate the heat shock response, crucial for coping with cellular stress, diminishes. This results in the accumulation of toxic misfolded proteins and diminished neuronal resilience (Broadley and Hartl, 2009; Trivedi et al., 2023; Zia et al., 2021). Dysregulated HSF-1, a master regulator of HSPs, has been explicitly linked to many age-related neurodegenerative diseases, including Alzheimer's and Huntington's Disease, where HSF-1, Hsp70, and Hsp90 proteins are expressed at low levels, mimicking normal aging processes (Figure 2) (Li J. et al., 2017; Trivedi et al., 2023).

The critical role of chaperones in aging and neurodegeneration is underscored by studies in various model organisms. In *Caenorhabditis elegans*, the upregulation of chaperones by HSF-1 and FOXO transcription factors is essential for extending lifespan (Hartl et al., 2011). Overexpression of Hsp70 in *Drosophila melanogaster* has been shown to completely suppress polyglutamine (polyQ) neurodegenerative disorders, lead to a twofold increase in lifespan, and reduce alpha-synuclein associated neuronal loss in Parkinson's disease models (Chaudhuri and Paul, 2006; Meriin and Sherman, 2005). Similarly, in mammalian models, Hsp70 overexpression proved effective against spinocerebellar ataxia (Chaudhuri and Paul, 2006). Modulating chaperone networks, whether through HSF-1 activation or the administration of exogenous HSPs, has demonstrated therapeutic potential by restoring proteostasis and improving cognitive performance in aging models (Li et al., 2017). For instance, intranasal administration of exogenous recombinant human Hsp70 significantly enhanced the lifespan of middle-aged and old mice and improved learning and memory (Albinhassan et al., 2025; Bobkova et al., 2015). This approach also mitigated Alzheimer's-like abnormalities in mouse models, reducing amyloid-beta accumulation and protecting spatial memory (Bobkova et al., 2013). Pharmacological interventions, such as Hsp90 inhibitors, can indirectly induce Hsp70, further highlighting therapeutic avenues for targeting chaperone pathways to counteract age-related proteotoxicity and neurodegeneration (Lackie et al., 2017). Therefore, maintaining or restoring robust Hsp70 and Hsp90 function could be a viable strategy to ameliorate the impact of age-related declines in proteostasis and prevent the onset or progression of neurodegenerative diseases (Hinault et al., 2006). Conversely, an age-related decline in proteostasis, characterized by reduced proteasomal activity and diminished chaperone capacity, significantly accelerates protein misfolding and aggregation, thereby contributing to neurodegeneration (Roe et al., 2018; Singh et al., 2025a,b). This age-dependent decline in chaperone efficacy, particularly for HSF-1, Hsp70, and Hsp90, creates a vulnerable cellular environment where the accumulation of toxic protein aggregates is exacerbated, directly linking aging processes to neurodegenerative pathologies (Broadley and Hartl, 2009; Gupta et al., 2019; Trivedi et al., 2023).

Building upon the detailed understanding of the role of Hsp70 and Hsp90 in aging, we can now delve into their crucial and multifaceted roles in specific neurodegenerative diseases. The subsequent sections will meticulously explore how these molecular chaperones, through their intricate mechanisms and interactions with co-chaperones, contribute to the pathogenesis and potential therapeutic intervention in conditions such as Alzheimer's disease.

## Hsp70 and Hsp90 in Alzheimer's disease

In Alzheimer's disease (AD), pathological hallmarks include the extracellular deposition of amyloid-beta plaques and intracellular accumulation of neurofibrillary tangles composed of hyperphosphorylated tau protein (Lackie et al., 2017; Ou et al., 2014). The brains of AD patients have higher levels of heat shock proteins, including Hsp27, Hsp70, and Hsp90, suggesting that these proteins are involved in the cellular reaction to protein misfolding stress (Koopman et al., 2020). According to Singh et al. (2025a),b, Hsp70 and Hsp90 play a crucial role in controlling the aggregation of pathogenic proteins, which in turn affects the course of disease (Singh et al., 2025a,b; Wei et al., 2024). By promoting both refolding and degradation pathways, Hsp70, for example, is crucial for containing amyloid-beta and other unfolded or misfolded proteins and reducing their deleterious effects. In order to help cells recover from stressors, this chaperone, together with Hsp90 can also control HSF-1, a master transcriptional regulator of the heat shock response (Evans et al., 2006; Lackie et al., 2017). Their coordinated action helps maintain cellular proteostasis, which is often disrupted in neurodegenerative conditions. For example, they can facilitate the clearance of misfolded proteins through autophagy, which is frequently compromised in Alzheimer's disease, or facilitate the ubiquitination of these proteins for proteasomal degradation (Barmaki et al., 2023).

Understanding the precise mechanisms through which Hsp70 and Hsp90 affect tauopathy, which include complex interactions with multiple co-chaperones and ATP-dependent cycle, is essential for determining their potential as therapeutic agents (Dixit et al., 2012). Hsp70, for example, serves as a flexible “multiple socket,” offering a site for the binding of co-chaperones, client proteins, and other cellular constituents, ultimately determining the client protein's fate within a particular cellular context (Salvatore et al., 2015). Hsp70 and its co-chaperones interact to determine whether tau or amyloid-beta is refolded, disassembled, or targeted for degradation, which has a significant effect on how proteotoxicity develops in Alzheimer's disease. In particular, Hsp70 has been shown to directly disrupt the formation of amyloid-beta aggregates and facilitate the proteasomal system's breakdown of tau and amyloid-beta oligomers (Valle-Medina et al., 2025). A complex interplay in tauopathy is highlighted by studies showing that the Hsp70 family, including constitutively expressed Hsc70, can both promote and inhibit tau degradation based on particular chaperone and co-chaperone interactions (Kundel et al., 2018; Miyata et al., 2011; Young et al., 2016). On the other hand, as a “master chaperone,” Hsp90 collaborates with many co-chaperones to regulate the folding, stability, and functionality of numerous proteins, many of which are transcription factors and kinases critical to cellular signaling pathways (Dixit et al., 2012). By guiding these abnormal proteins toward degradation pathways like the ubiquitin-proteasome system and chaperone-mediated autophagy, Hsp90 cooperates with Hsp70 in the context of the cellular response to protein misfolding, especially with regard to amyloid-beta plaques and tau tangles (Cavanaugh et al., 2015). Furthermore, Hsp90, together with Hsp70, control the activity of important enzymes involved in tau phosphorylation and dephosphorylation, which helps to prevent the development of neurofibrillary tangles (Dixit et al., 2012). Hop, a co-chaperone that serves as an adaptor linking Hsp70 and Hsp90, was reported to facilitate the transfer of client proteins between these two major chaperone systems and modulate their collaborative efforts in protein quality control (Schmidt et al., 2015). Indeed, co-chaperones that alter Hsp90's ATPase activity frequently mediate this regulation, affecting its binding affinity for client proteins such as tau and the kinases that phosphorylate it (Pearl, 2016; Prodromou, 1999).

By affecting tau's phosphorylation state and subsequent degradation pathways, the complex network of Hsp90 and its co-chaperones, such as FKBP51 and FKBP52, is essential for preserving tau proteostasis (Jeanne et al., 2024; Jinwal et al., 2010). These immunophilins, in particular FKBP51, have the ability to alter tau's conformational dynamics and its binding to Hsp90, which in turn affects tau phosphorylation and aggregation (Jeanne et al., 2024). Conversely, inhibition of Hsp90 has been shown to reduce tau levels, suggesting that while chaperones generally support protein folding, their dysregulation or excessive activity under pathological conditions can contribute to neurodegeneration (Goryunov and Liem, 2007; Luo et al., 2010). This intricate interaction demonstrates the paradoxal role of Hsp90 in tau pathology, as its interplay with different co-chaperones determines whether tau is stabilized and allowed to aggregate or destabilized and degraded (Shelton et al., 2017).

Since tau hyperphosphorylation and subsequent aggregation are essential for the formation of neurofibrillary tangles, the complex interaction between molecular chaperones and tau protein aggregation is a central focus of research on Alzheimer's disease. With its varied co-chaperone complexes, Hsp90 in particular has a multifaceted role in controlling tau pathology (Shelton et al., 2017). These complexes can either promote tau degradation or, paradoxically, stabilize pathogenic tau species. In particular, post-translational modifications of tau protein, such as phosphorylation, ubiquitination, and acetylation, significantly impact how it interacts with molecular chaperones, which in turn determines its propensity for neurotoxicity and aggregation. Whether tau is correctly folded and functional or misfolded, aggregated, and neurotoxic is ultimately determined by this oscillation between tau modifications and chaperone engagement (Kontaxi et al., 2017; Miyata et al., 2011; Peak et al., 2020). According to recent studies, certain alterations can change tau's typical role of stabilizing microtubules and contribute to its pathobiology, which increases the protein's propensity to aggregate (Kadavath et al., 2015). Furthermore, hyperphosphorylated tau has lower affinity for microtubules and is more likely to detach, which increases the likelihood of self-association and fibril formation (Sun and Gamblin, 2009). Interestingly, specific post-translational modifications of tau have a significant effect on Hsp90's affinity for tau protein, underscoring its critical role in the pathophysiology of Alzheimer's disease (Salminen et al., 2010; Shelton et al., 2017). In particular, it has been demonstrated that FKBP51 increases tau phosphorylation and aggregation by altering how Hsp90 interacts with client proteins, whereas Aha1, a Hsp90 co-chaperone, can increase Hsp90's ATPase activity, which in turn affects tau client processing and possibly its degradation (Jeanne et al., 2024; Jinwal et al., 2010). In addition, certain phosphorylation sites on tau are recognized by chaperones such as Hsp27, Hsp70, and CHIP, which can then facilitate its degradation or dephosphorylation (Sabbagh et al., 2016). In addition, a key player in triggering tau ubiquitination, which promotes tau degradation and inhibits aggregation, is CHIP, a ubiquitin ligase that interacts directly with Hsp70/90 (Munari et al., 2022). With the help of Hsp90 chaperone complexes, CHIP activity can control the proteasomal and autophagic degradation pathways of tau protein, suggesting a finely tuned mechanism for protein quality control (Dickey et al., 2007; Munari et al., 2022). On the other hand, abnormalities in CHIP expression or function are linked to the buildup of misfolded tau, which aids in the development of tauopathies (Saidi et al., 2015). A direct connection between kinase activity and chaperone-mediated protein turnover in AD is also suggested by the interaction between Akt and the Hsp90/CHIP complex, which has been demonstrated to affect tau ubiquitination and subsequent degradation (Dickey et al., 2008).

Moreover, direct binding of Hsp90 to tau induces a conformational shift that promotes aggregation, underscoring the need to selectively disrupt this interaction rather than globally inhibit Hsp90 activity (Karagöz et al., 2014). Consequently, therapeutic approaches are focusing on selectively disrupting pathogenic Hsp90-CHIP assemblies or enhancing Hsp70-BAG2-mediated tau delivery to the proteasome, thereby promoting clearance of misfolded tau while preserving essential chaperone functions (Carrettiero et al., 2009). This duality highlights the regulatory mechanisms required for chaperone-based therapeutic interventions (Salminen et al., 2010). For example, research shows that Hsp90 inhibitors can lower phosphorylated tau levels, indicating that preventing the folding or refolding pathway encourages the breakdown of tau protein that has been abnormally altered. This observation is consistent with research showing that Hsp90 inhibition promotes phospho-tau clearance mainly through proteasomal degradation, with a minimal lysosomal pathways contribution (Dickey et al., 2007). On the other hand, the development of pathological chaperone complexes called epichaperomes can promote tau aggregation and is linked to the advancement of Alzheimer's disease. This suggests that a more focused therapeutic approach might be to target these aberrant assemblies rather than global chaperone function (Salminen et al., 2010). Recent drug discovery efforts have identified Hsp90-selective inhibitors that destabilize pathogenic tau while sparing Hsp70-mediated protective pathways, offering a refined strategy for modulating proteostasis in AD (Salminen et al., 2010).

The age-related decline in chaperone activity significantly amplifies tau pathology, a central feature in Alzheimer's disease and other tauopathies. As the brain ages, the expression and function of essential heat shock proteins like Hsp70 and Hsp90 decrease, impairing the cell's ability to manage misfolded and aggregated proteins (Dickey et al., 2007; Hervás and Oroz, 2020; Shelton et al., 2017). This diminished proteostasis capacity creates an environment where tau becomes more susceptible to hyperphosphorylation and aggregation, ultimately leading to the formation of neurofibrillary tangles (Blair, 2014; Tangavelou and Bhaskar, 2024). Specifically, changes in the Hsp90 heterocomplex, including increased levels of co-chaperones like FKBP51 and Aha1 can promote tau accumulation and neurotoxicity in aged brains, while protective chaperones may be reduced (Bohush et al., 2019; Criado-Marrero et al., 2021; Hervás and Oroz, 2020; Shelton et al., 2017). These age- and disease-related shifts in the cellular chaperone repertoire directly contribute to the pathological buildup of tau (Criado-Marrero et al., 2020).

The delicate balance of the chaperone network is crucial for maintaining tau solubility and preventing its aggregation (Dou et al., 2003). However, with advancing age, this balance is disrupted. For instance, the age-dependent increase in Cdc37, an Hsp90 co-chaperone, has been linked to alterations in tau phosphorylation, which can increase its toxicity and reduce its stability (Bohush et al., 2019). Conversely, a reduction in the activity of phosphatases, which are also Hsp90 co-chaperones, in an aging or AD brain can contribute to tau hyperphosphorylation (Bohush et al., 2019). While Hsp90 typically plays a role in stabilizing tau, its activity under pathological conditions can sometimes promote tau aggregation, highlighting a complex and potentially paradoxical role that is influenced by co-chaperones and the overall aging environment (Blair, 2014; Singh et al., 2025a,b). Therefore, the age-associated decline and imbalance within the Hsp70/Hsp90 chaperone machinery are critical factors in the exacerbation of tau pathology, making the understanding and modulation of these chaperones an important therapeutic strategy for age-related neurodegenerative diseases (Blair et al., 2013; Shelton et al., 2017).

## Hsp70 and Hsp90 in Parkinson's disease

Parkinson's disease (PD) is characterized by a core pathological feature: proteinopathy driven by the misfolding and aggregation of alpha-synuclein, which leads to the formation of toxic oligomers and insoluble Lewy bodies (Sidoryk-Wȩgrzynowicz et al., 2024). Molecular chaperones play an essential role in counteracting this proteotoxicity by modulating the accumulation of these aberrant protein species. Specifically, heat shock proteins, including Hsp70 and Hsp90, are crucial in mitigating alpha-synuclein pathology by promoting its proper folding or facilitating its degradation and clearance (Li et al., 2024). Furthermore, small heat-shock proteins, such as Hsp27, contribute by binding to early alpha-synuclein intermediates, thereby impeding fibril elongation (Cox et al., 2018; Jia et al., 2019). Consequently, neuronal survival hinges on the proper functioning of these chaperone systems; their overload or malfunction significantly influences the development of neurodegeneration in Parkinson's disease. Hence, restoring proteostasis by modifying chaperone activity could constitute a promising therapeutic strategy for preventing the buildup of toxic protein species in Parkinson's disease.

Hsp70 actively interacts with misfolded alpha-synuclein in PD, promoting its proteasomal breakdown. This direct action of Hsp70 aids in preventing the buildup of toxic alpha-synuclein species and their subsequent aggregation into Lewy bodies, which are disease-defining features (Choudhary et al., 2025; Li et al., 2024). The preservation of neuronal proteostasis, therefore, critically depends on Hsp0's capacity to either target alpha-synuclein for degradation or encourage its refolding. Experimental evidence further highlights Hsp0's protective ability, showing that overexpressing the human protein significantly reduces dopaminergic neuron loss in models such as *Drosophila* (Li et al., 2024; Shukla et al., 2014)

Conversely, the role of Hsp90 is characterized by a dynamic balance. Its activity is essential for stabilizing soluble conformers of alpha-synuclein, and its intricate interaction within the proteostasis network also presents therapeutic opportunities. For instance, in rodent models, it has been demonstrated that pharmacologically inhibiting cytosolic Hsp90 causes a compensatory expression of Hsp70, which subsequently actively lowers alpha-synuclein toxicity (Putcha et al., 2009). By increasing Hsp70 levels, brain-penetrant Hsp90 inhibitors can inhibit the formation of alpha-synuclein oligomers and restore striatal dopamine. This suggests that while Hsp90 can stabilize alpha-synuclein, manipulating its activity can indirectly induce clearance mechanisms mediated by Hsp70. Indeed, pharmacologically inducing Hsp70, either through Hsp90 inhibition or direct activators, has been shown to lower α-synuclein oligomer levels and protect dopaminergic neurons (Tao et al., 2021). Thus, Hsp90, while stabilizing soluble forms, also offers a pathway for therapeutic intervention by indirectly upregulating Hsp70.

The effectiveness of both Hsp70 and Hsp90 in managing alpha-synuclein strongly depends on their co-chaperones interaction network that fine-tunes their chaperone activities. For example, CHIP, an E3 ubiquitin ligase and a major Hsp70' and Hsp90's interacting co-chaperone, functions as an directly promoting alpha-synuclein's proteasomal and lysosomal degradation (Tetzlaff et al., 2008). Hip, another co-chaperone, is essential for maintaining the stability of the Hsp70/alpha-synuclein complex, especially when Hsp70 is bound to ADP, thereby preventing aggregation. Studies indicates that lower Hip levels in early-stage Parkinson's disease patients hinder Hsp70's capacity to inhibit alpha-synuclein aggregation, underscoring the importance of co-chaperone balance (Roodveldt et al., 2009). Thereby, Hsp0's ability to regulate alpha-synuclein may be improved by restoring or increasing Hip levels. Conversely, small Heat-Shock Proteins attach to early alpha-synuclein intermediates, which slows down the growth of fibrils and acts as a first line of defense against aggregation (Roodveldt et al., 2009). Hsp27, α- and β-crystallin markedly reduce α synuclein aggregation and neurotoxicity in dopaminergic neurons, highlighting the therapeutic potential of small heat shock proteins (Cox et al., 2018). Therefore, strategies that increase the levels of Hsp27 or improve its interaction with α-synuclein are being investigated as potential disease-modifying interventions in Parkinson's models. In conclusion, the intricate interplay with co-chaperones ensures the specificity and efficiency of these chaperone activities, making the entire chaperone network a critical determinant in the progression of Parkinson's disease (Cox et al., 2018).

The decline in chaperone activity with age also profoundly impacts dopaminergic neurons, contributing to their vulnerability in age-related neurodegenerative conditions like Parkinson's disease. As the brain ages, the activity and availability of critical molecular chaperones, including Hsp70, generally decrease in neuronal tissue (Bobkova et al., 2015). This reduction in chaperone capacity leaves dopaminergic neurons less equipped to handle the burden of misfolded proteins and cellular stress, making them particularly susceptible to damage and degeneration (Bonini, 2002). This diminished proteostasis can be seen as a key factor in the onset and progression of dopaminergic neuron pathology, as the cells struggle to maintain protein quality control in the face of accumulating aberrant protein species. Indeed, studies have shown a significant reduction in the expression of Hsc70, a constitutively expressed Hsp70 isoform, in the substantia nigra of individuals with Parkinson's disease, a region critically affected by dopaminergic neuron loss (Fishman-Jacob and Youdim, 2023). Conversely, experimental upregulation of Hsp70 has demonstrated neuroprotective effects, reducing alpha-synuclein aggregation and toxicity, and promoting the survival of dopaminergic neurons in various models (Ribeiro et al., 2021; Singh et al., 2025a,b; Wankhede et al., 2022). While some research suggests a compensatory increase in Hsp72 and Hsc70 in specific brain regions, like the substantia nigra, during aging in rats, the overall functional capacity to effectively manage proteotoxic stress appears compromised, ultimately failing to prevent neurodegeneration (Calabrese, 2004). This age-related reduction in the functional Hsp70 chaperone system thus plays a critical role in increasing the susceptibility of dopaminergic neurons to misfolding protein stress and neurodegeneration.

## Hsp70 and Hsp90 in amyotrophic lateral sclerosis and Huntington's disease

Amyotrophic Lateral Sclerosis (ALS) and Huntington's Disease (HD) are distinguished by unique proteinopathies that facilitate neurodegeneration. In ALS, a major hallmark is the accumulation of TDP-43, while in HD, the aggregation of mutant huntingtin protein within neurons constitutes the main (Watanabe et al., 2020). These misfolded proteins combine to form toxic aggregates that impair cellular processes and cause neurons dysfunction and eventual death. As an essential line of defense against proteotoxicity, molecular chaperones, which are essential parts of the cellular proteostasis network, control these abnormal protein species by ensuring proper folding, refolding stress-damaged proteins, and avoiding aggregate formation (Miller and James, 2016).

In the context of ALS and HD, the Hsp70 and Hsp90 chaperone families counteract the pathological processes initiated by misfolded TDP-43 and mutant huntingtin (García-Toscano et al., 2024; Lin et al., 2021). These chaperones facilitate correct folding or target misfolded proteins for degradation via the ubiquitin-proteasome system and autophagy, thereby playing a pivotal role in maintaining neuronal proteostasis and delaying disease progression by mitigating aggregate formation and accumulation. Their robust activity is essential for neuronal survival; any compromise contributes significantly to disease pathology, making chaperone modulation a promising therapeutic avenue (Batko et al., 2024). Pharmacological inhibition of Hsp90, which concomitantly up-regulates Hsp70 and Hsp40, represents a promising therapeutic modality to accelerate mutant huntingtin and TDP-43 aggregate removal in ALS and Huntington's disease models (Luo et al., 2010). Additionally, induction of Hspb8, which cooperates with BAG3 and the Hsp70/HSC70-CHIP complex to stimulate autophagic removal of misfolded TDP-43, represents a complementary avenue to increase proteostatic capacity in ALS and related neurodegenerative conditions (Crippa et al., 2010; Cristofani et al., 2019). Furthermore, inducing the small-heat-shock protein HspB8 to engage BAG3-mediated autophagy markedly improves clearance of TDP-43 aggregates and attenuates neurodegeneration in ALS models (Crippa et al., 2010). Collectively, these findings highlight that simultaneous activation of Hsp70/Hsp40 and HSPB8-BAG3 autophagic pathways synergistically restores proteostasis and offers a robust strategy to counteract TDP-43 and mutant huntingtin toxicity, establishing a mechanistic rationale for combined therapeutic approaches (Koay et al., 2014; Lackie et al., 2017; Wei et al., 2024; Harding and Tong, 2018). Future investigations should assess combinatorial strategies that couple selective Hsp90 inhibition with HspB8-mediated autophagy to achieve durable restoration of proteostasis in ALS and Huntington's disease. Notably, the Hsp70/Hsp40 machinery directly engages mutant huntingtin, facilitating its lysosomal delivery via Hsc70 and concurrently recruiting the CHIP-Hsp70 E3 ligase axis to ubiquitinate and clear toxic species, thereby reinforcing proteostatic resilience in HD models (Lotz et al., 2010). Emerging pharmacological agents, including SNX-derived Hsp90 inhibitors that also activate HSF-1 and up-regulate Hsp70, may further amplify the synergistic benefit of combined Hsp90 blockade and HspB8-mediated autophagy, advancing these strategies toward clinical translation (Mathenjwa et al., 2024; Steurer et al., 2022; Wang et al., 2014).

Moreover, activation of the heat-shock response to up-regulate Hsp70 has been shown to facilitate proteasomal degradation of mutant huntingtin and TDP-43, supporting a synergistic approach alongside isoform-specific targeting (Chen et al., 2016). Importantly, preclinical validation of combined Hsp90 inhibition with HSPB8-mediated autophagy in animal models will be essential to confirm synergistic efficacy and safety before clinical translation (Chen et al., 2020; Han et al., 2018; Li et al., 2017). Future work should delineate the isoform-specific contributions of Hsp70 family members, such as HspA5, to TDP-43 and mutant huntingtin clearance, as recent evidence indicates direct binding of Grp78/BiP to TDP-43 mitigates toxicity (Chen et al., 2016; Pinho et al., 2021).

In motor neurons, this age-dependent decrease in chaperone capacity means that these vital cells are less capable of initiating a robust cytoprotective heat shock response when confronted with increasing amounts of misfolded proteins, a key characteristic of ALS (Gil et al., 2017). Aging is marked by a decline in the overall functioning of heat shock proteins and protein degradation machinery, leading to an increased inability to effectively refold or clear misfolded proteins (Qu et al., 2018). For example, the age-related impairment of Hsp induction critically contributes to the vulnerability of motor neurons, particularly in diseases like Amyotrophic Lateral Sclerosis. This phenomenon is compounded by the fact that senescent cells often exhibit lower chaperone protein translation (Venediktov Artem et al., 2023). In addition, HSF-1appears to have a relatively high threshold for activation in motor neurons, and even the presence of disease-associated misfolded proteins, such as mutant SOD1, may not be sufficient to trigger adequate HSF-1 activation or Hsp induction (Gil et al., 2017). This inherent limitation in the stress response machinery leaves motor neurons particularly susceptible to the accumulation of toxic protein aggregates, thereby accelerating their degeneration. In addition, this compromised Hsp induction in aging motor neurons directly exacerbates the pathology seen in ALS. Studies have shown that in motor neurons overexpressing mutant SOD1, there is generally no detectable basal expression or upregulation of key Hsps, with the notable exception of Hsp27, which itself progressively declines with disease advancement (Gil et al., 2017). The high threshold for HSF-1 activation in motor neurons contributes significantly to their vulnerability and limits the efficacy of therapeutic agents aimed at increasing Hsp expression (Batulan et al., 2006). Conversely, augmenting the HSR through interventions like the overexpression of HSF-1 has demonstrated neuroprotective effects, improving motor function and survival rates while decelerating motor neuron degeneration in ALS models (Xu et al., 2025). While pharmacological upregulation of Hsps can rescue motor neurons from cell death in ALS models, the relationship is complex, indicating the nuanced role of these chaperones in disease progression (Kalmár and Greensmith, 2009). The decline in neuroprotective Hsps, including specific small heat shock proteins like Hsp27 and HspB8, which are linked to motor neuron neuropathies (Li X. et al., 2017), underscores the importance of maintaining robust chaperone activity to protect these vulnerable neuronal populations from age-related proteotoxic stress (Singh et al., 2025a,b).

## Therapeutic potential of targeting Hsp70 and Hsp90 proteins

Traditional investigation of neurodegenerative diseases often focuses on selected protein aggregates or isolated cellular dysfunctions without considering the entire proteostasis network. Disruptions within this network, rather than mere deficiencies of individual chaperones, are now considered key drivers in the onset and progression of these disorders. Prioritizing the restoration of overall proteostasis could therefore strengthen cellular defenses against the buildup of misfolded proteins and lead to advanced therapeutic strategies that rebalance the proteostasis machinery, preventing aberrant protein aggregation. To develop more targeted and effective therapies for neurodegeneration, the precise mechanisms by which chaperones recognize, and process misfolded proteins should be elucidated.

Molecular chaperones, particularly Hsp70 and Hsp90, are now recognized as more than just protein-folding assistants. Their roles encompass complex regulatory functions within cellular proteostasis networks, vital for maintaining cellular equilibrium through an interplay of the ubiquitin-proteasome system, autophagy pathways, and various co-chaperones (Fernández-Fernández et al., 2017; Hu et al., 2022; Somogyvári et al., 2022). Disease-specific roles of Hsp70, Hsp90, and associated co-chaperones across major neurodegenerative disorders are summarized in Table 2. This extensive involvement makes Hsp70 and Hsp90 promising therapeutic targets in neurodegeneration, as their modulation can effectively prevent proteotoxic stress.

**Table 2:** Disease-specific roles of Hsp70 and Hsp90 in major neurodegenerative disorders.

**Disease**	**Major pathogenic protein(s)**	**Hsp70 role**	**Hsp90 role**	**Key co-chaperones involved**	**Net effect**
Alzheimer's disease (AD)	Tau, Aβ	Promotes refolding and degradation of misfolded tau; suppresses tau aggregation and toxicity	Stabilizes pathogenic tau species; excessive Hsp90 activity sustains tau pathology	CHIP, BAG1/3, FKBP51, Aha1	Context-dependent (Hsp70 protective; Hsp90 often pathogenic when dysregulated)
Parkinson's disease (PD)	α-Synuclein	Inhibits oligomer formation; enhances clearance via proteasome and autophagy	Can stabilize α-synuclein oligomers; inhibition reduces toxicity	Hip, Hsp40, CHIP	Protective when Hsp70 dominant
Amyotrophic lateral sclerosis (ALS)	TDP-43, SOD1, FUS	Facilitates clearance of misfolded proteins; supports stress resilience	Dysregulated Hsp90 contributes to persistence of toxic clients	BAG3, HspB8, Sti1	Predominantly protective
Huntington's disease (HD)	Mutant huntingtin (mHTT)	Suppresses aggregation and promotes degradation of soluble mHTT	May buffer misfolded mHTT but also stabilize toxic conformers	Hsp40, CHIP, BAG3	Protective with limits
Age-related proteostasis decline	Multiple	Reduced inducibility and efficiency with aging	Decreased regulatory control and altered co-chaperone balance	HSF-1, BAG family	Progressive loss of protection

Therapeutic strategies directly targeting Hsp70 and Hsp90 activity are currently being explored to mitigate neurodegenerative diseases. For instance, Hsp90 inhibitors have shown promising results in preclinical models by promoting the degradation of misfolded tau and amyloid-beta species, thereby alleviating proteotoxicity and offering a novel therapeutic avenue for Alzheimer's disease (Ou et al., 2014). These inhibitors function by interfering with the interactions between Hsp90 and its client proteins, leading to ubiquitination and subsequent proteasomal degradation (Park et al., 2019). Additionally, molecules such as rhodacyanine derivatives and phenothiazines have been demonstrated to lower tau levels by inhibiting Hsp70 ATPase activity, underscoring the therapeutic potential of directly modulating chaperone function (Hill et al., 2023; Martin et al., 2016; Shao et al., 2021).

Another promising class of therapeutic agents includes pharmacological chaperones, which bind and stabilize misfolded proteins to promote proper folding and trafficking. By restoring native conformation and reducing aggregation, these compounds can effectively counteract neurodegenerative processes. Moreover, the development of small-molecule modulators and co-chaperone inhibitors, particularly those that target protein–protein interfaces between chaperones and their regulatory partners, represents a rapidly advancing therapeutic avenue (Wang et al., 2021). For instance, while some Hsp90 inhibitors targeting the N-terminal ATP binding site can lead to broad inhibition and adverse effects, newer strategies focus on the Hsp90 C-terminus with compounds like dihydropyridine derivatives. Such compounds can selectively modulate co-chaperone interactions without triggering a detrimental heat shock response, thereby showcasing neuroprotective potential (Lu et al., 2008; Roe et al., 2018; Wang et al., 2021).

Beyond direct chaperone inhibition, other strategies focus on modulating co-chaperones like FKBP51 and Aha1 to indirectly control Hsp90 activity and alleviate tau pathology (Gu et al., 2025; Jeanne et al., 2024; Oroz et al., 2019). FKBP51, the Hsp90 co-chaperone, is implicated in tau pathology, with elevated levels promoting tau accumulation and neurotoxicity. Consequently, downregulation of FKBP51 or selective inhibitors of its PPIase activity can reduce tau levels, highlighting its potential as a therapeutic target (Blair et al., 2014, 2013; Criado-Marrero et al., 2021; Kumar et al., 2017). Another example, Aha1, a potent accelerator of Hsp90 ATPase activity, when inhibited, can lead to decreased formation of tau and Aβ aggregates (Blagg and Catalfano, 2024; Bohush et al., 2019). Similarly, upregulating the expression of different chaperones through the induction of the heat shock response, a cellular defense mechanism, also provides a broad-spectrum approach to bolster cellular proteostasis, which can be achieved through pharmacological agents or gene therapy (Blair et al., 2013; Chakraborty and Zweckstetter, 2025; Jinwal et al., 2010).

The BAG family of co-chaperones, encompassing BAG1, BAG2, and BAG3, also represents a critical target for small-molecule modulation. This is due to their pivotal role as nucleotide exchange factors for Hsp70 and their involvement in crucial protein degradation pathways (Broadley and Hartl, 2009; Gu et al., 2025). For instance, BAG2, in complex with Hsp70, can promote the proteasomal degradation of tau in a ubiquitin-independent manner and prevent tau hyperphosphorylation (Carrettiero et al., 2009; Lima et al., 2022; Yang et al., 2023). BAG3 is integral to macroautophagy, linking Hsp70 to this process for the clearance of misfolded proteins and collaborating with small heat shock proteins like HSPB8 (Broadley and Hartl, 2009; Chierichetti et al., 2022; Kaushik and Cuervo, 2012; Vendredy et al., 2020). Its upregulation has been shown to facilitate tau clearance in neurons and is implicated in various neurodegenerative diseases, including Huntington's, ALS, Parkinson's, and Alzheimer's (Kirk et al., 2021; Lei et al., 2014; Ying et al., 2022). Small molecules such as YM-1 and JG-98 have been developed to disrupt Hsp70-BAG3 interactions. Analogs like JG-48 and YM-8 have demonstrated the ability to increase tau turnover and reduce tau phosphorylation in brain slices, and critically, they can penetrate the blood-brain barrier (Gu et al., 2025; Wang et al., 2021). Furthermore, BAG6 has been identified for its role in preventing the aggregation of TDP43 fragments associated with neurodegeneration (Kasu et al., 2022). This targeted modulation of BAG family members thus underscores the multifaceted strategies being explored to restore proteostasis and combat neurodegeneration.

Experimental administration of recombinant heat shock proteins has also emerged as a promising neuroprotective strategy, offering a direct means to bolster the cellular chaperone machinery against proteotoxic stress (Demyanenko et al., 2020, 2023; Shevtsov et al., 2014). In various preclinical models, recombinant Hsp70 has demonstrated significant therapeutic potential. For instance, in rodent models of cerebral ischemia, direct administration of rHsp70ex led to a remarkable twofold reduction in infarct volume, thereby mitigating local ischemia in the prefrontal brain cortex (Demyanenko et al., 2020). This neuroprotective effect was accompanied by a decrease in apoptosis within the ischemic penumbra, stimulation of axonogenesis, and an increase in synaptophysin-producing neurons (Demyanenko et al., 2020). Furthermore, mice overexpressing Hsp70 subjected to middle cerebral artery occlusion exhibited reduced infarct size and improved neurological deficits, alongside a decrease in activated microglia and macrophages in ischemic brain regions (Zheng et al., 2007). Beyond stroke models, intranasally administered Hsp70 has been shown to rapidly reach affected brain areas, alleviating Alzheimer's-like morphological and cognitive impairments, including reduced amyloid-beta accumulation and preserved spatial memory (Bobkova et al., 2013). Similarly, studies focusing on alpha-synuclein pathology have revealed that Hsp70 overexpression reduces insoluble alpha-synuclein aggregates and levels, also decreasing associated neuronal toxicity, ultimately promoting the survival of dopaminergic neurons in transgenic mouse and Drosophila melanogaster models (Wankhede et al., 2022). Recombinant human Hsp70 has also been shown to increase lifespan, delay symptom onset, preserve motor function, and prolong motor neuron survival in a mouse model of Amyotrophic Lateral Sclerosis (Gifondorwa et al., 2007). While the therapeutic potential of recombinant Hsp90, particularly its protective immune-modulatory effects in models of spinal cord injury and Amyotrophic Lateral Sclerosis, is an active area of interest, direct evidence specifically demonstrating these precise effects from recombinant Hsp90 protein administration is not widely documented. Nonetheless, related research indicates Hsp0's broader involvement in influencing immune responses and proteostasis in these conditions. For example, extracellular Hsp90α has been observed to stimulate innate immune responses in microglial cells, characterized by the activation of NF-kB-regulated genes, and to protect against oxidative stress (Okusha et al., 2022), suggesting a potential immune-modulatory function. Moreover, the therapeutic exploration of Hsp90 often involves its modulation, which in ALS models has been shown to decrease neurotoxicity (Lee et al., 2023). Other recombinant heat shock proteins, such as small HspB1, have demonstrated anti-inflammatory effects in astrocytes, reducing inflammatory markers and cytokine secretion, which highlights the general strategy of using recombinant chaperones for immune modulation (Yang et al., 2024). These findings collectively underscore the complex involvement of Hsp90 and other chaperones in neuroinflammation and proteostasis, indicating that further specific investigation into the direct immune-modulatory effects of recombinant Hsp90 in SCI and ALS models may be warranted.

Given the complex and multifaceted nature of neurodegenerative diseases, a monotherapeutic approach may not be sufficient to address such conditions. Consequently, combination therapies that target several different chaperone pathways or combine chaperone modulation with other therapeutic approaches, like those that target mitochondrial dysfunction, may be more effective. The development of highly specific and selective modulators for distinct chaperone isoforms should be a priority of future research to enable targeted interventions with few off-target effects. Furthermore, a significant advancement is represented by personalized medicine strategies that customize treatment plans to address the unique proteostasis deficiencies found in each patient. Lastly, the discovery of biomarkers that can indicate the integrity of the proteostasis network *in vivo* will facilitate monitoring treatment efficacy and develop more personalized treatment plans.

## Conclusion

In conclusion, this review extends beyond previous syntheses by integrating the molecular biology of Hsp70 and Hsp90 with the gradual loss of chaperone capacity that occurs with aging, as well as exploring translational therapeutic strategies involving recombinant Hsp interventions and isoform-level mechanisms. This comprehensive approach highlights that targeting Hsp70 and Hsp90 presents a promising avenue to transform the landscape of neurodegenerative disease treatment.

By adopting a comprehensive and personalized approach that addresses the complex interactions of the proteostasis network, we can unlock novel therapeutic targets and strategies, ultimately moving beyond symptomatic relief toward effective disease modification and prevention. Future directions will focus on the precision modulation of individual Hsp isoforms, alongside the development of combination therapies that integrate HSF-1 activation, recombinant Hsp delivery, and broader proteostasis enhancement. By further deciphering the intricacies of chaperone biology and utilizing cutting-edge therapeutic approaches, we can pave the way for more effective interventions against these devastating conditions. However, despite the promising results from targeting Hsp70 and Hsp90, the complex interplay of a vast array of co-chaperones and the existence of natural polymorphisms necessitate a deeper understanding of the entire proteostasis network for developing strategic therapeutic approaches.
